# Phenytoin-Induced Severe Thrombocytopenia: A Case Report

**DOI:** 10.7759/cureus.60669

**Published:** 2024-05-20

**Authors:** Taweesak Maneerot, Pirun Saelue, Antida Sangiemchoey

**Affiliations:** 1 Department of Clinical Pharmacy, Faculty of Pharmaceutical Sciences, Prince of Songkla University, Songkhla, THA; 2 Hematology Unit, Division of Internal Medicine, Faculty of Medicine, Prince of Songkla University, Songkhla, THA; 3 Division of Pharmacy, Songklanagarind Hospital, Faculty of Medicine, Prince of Songkla University, Songkhla, THA

**Keywords:** phenytoin induced thrombocytopenia, antiepileptic drug, drug-induced thrombocytopenia, thrombocytopenia, phenytoin

## Abstract

Phenytoin is a commonly prescribed antiepileptic medication for the prevention and treatment of tonic-clonic or partial seizures. Thrombocytopenia is a rare and serious adverse effect of phenytoin. This case report presents the case of a patient with severe thrombocytopenia induced by phenytoin for the treatment of tonic-clonic seizures. A 63-year-old male received 300 mg/day of phenytoin for the treatment of tonic-clonic seizures. Seven days after receiving the first dose of phenytoin, he was diagnosed with severe thrombocytopenia (platelet count 44 x 10^9^/L) without hemorrhage. Phenytoin was discontinued, and seizures were controlled with levetiracetam. Seven days after stopping phenytoin, his daily platelet count improved from 44 to 177 x 10^9^/L. The Naranjo algorithm score of 7 was at a probable level for phenytoin-induced thrombocytopenia. Thrombocytopenia is a serious adverse drug reaction that can result in life-threatening bleeding. Phenytoin-induced thrombocytopenia commonly begins 1-90 days after administration, and the recovery time is 3-21 days. The potential mechanism of phenytoin-induced thrombocytopenia is drug-induced immune thrombocytopenia. Drugs that enhance the concentration of phenytoin epoxide may be a contributing factor in phenytoin-induced thrombocytopenia. Phenytoin-induced thrombocytopenia is a rare but serious hematological complication. It should be recognized early, particularly in patients with a high risk of hemorrhage or concurrently with medications that increase phenytoin epoxide. Regularly consecutive complete blood count tests may be essential in order to detect an early decrease in platelet count in these patients.

## Introduction

Drug-induced thrombocytopenia (DIT) is a relatively rare but severe adverse drug reaction [1]. Thrombocytopenia is defined as a platelet count of less than 150 x 10^9^/L [2]. Patients with severe thrombocytopenia (platelet count of less than 50 x 10^9^/L) can experience spontaneous bleeding such as ecchymoses, petechiae, mucosal bleeding, or life-threatening spontaneous intracranial hemorrhage [1]. The incidence rates of major and fatal hemorrhages in patients with severe thrombocytopenia were 9 and 0.8%, respectively [3].

In general, the platelet count decreases within seven days or more after beginning a new drug or 2-3 days after consuming a previously consumed drug. Within 1-10 days of stopping the drug, the platelet count rises to the normal range [1,3].

The incidence rates of the medications responsible for DIT vary [4]. The incidence of DIT among critically ailing patients is approximately 25%, whereas the overall incidence rate is approximately 10 cases per million persons per year [4]. Anticonvulsants, such as carbamazepine, phenytoin, and valproic acid, are among the drugs most frequently associated with DIT [5].

Phenytoin is a commonly prescribed antiepileptic medication for the prevention and treatment of tonic-clonic or partial seizures [4]. Thrombocytopenia is a rare and serious adverse effect of phenytoin [4]. In this article, we present a case of phenytoin-induced thrombocytopenia in a patient with a generalized tonic-clonic seizure.

## Case presentation

A 63-year-old male was admitted to the internal medicine ward for treatment of generalized tonic-clonic seizures. Four months prior to admission, he had an accident resulting in an epidural hematoma and cervical spondylosis cord compression. The craniotomy with clot removal was employed to manage an epidural hematoma. Long-term gabapentin and nortriptyline were prescribed for the treatment of cervical spondylosis and cord compression. Subsequently, he received a diagnosis of a syndrome of inappropriate antidiuretic hormone secretion and received a sodium chloride tablet for treatment of this condition. Three days before admission, he had watery diarrhea and confusion. Moreover, he overused sodium chloride tablets because of a misunderstanding.

On the first day of admission, his serum sodium was 257.2 mmol/L, and his complete blood count, liver function, and renal function were normal. He received a diagnosis of symptomatic hypernatremia and presented with generalized tonic-clonic seizures. The etiology of hypernatremia was suspected to be dehydration and overuse of sodium chloride tablets. Phenytoin, 100 mg IV every 8 hours, was prescribed to control seizures for one day. After that, phenytoin was changed from intravenous to phenytoin 50 mg tablet administration via nasogastric tube, two tablets every 8 hours (total daily dose: 300 mg/day).

Four days later, his serum sodium decreased into the normal range, but he still had a generalized tonic-clonic seizure. Therefore, phenytoin was continued, and levetiracetam 1000 mg IV every 12 hours was added to control seizures. Furthermore, he was diagnosed with ventilator-associated pneumonia. He received ceftazidime for treatment of this condition.

Seven days after receiving the first dose of phenytoin, his platelet count decreased from 539 to 44 x 10^9^/L. He had no signs or symptoms of bleeding. The peripheral blood smear showed thrombocytopenia without platelet aggregation and normal red blood cell morphology. Consequently, a multidisciplinary team worked together to find the cause of this patient's thrombocytopenia, such as hypersplenism due to chronic liver disease, chronic alcohol abuse, nutrient deficiencies (folate and vitamin B12), autoimmune disorders (systemic lupus erythematosus and rheumatoid arthritis), and lymphoproliferative diseases (non-Hodgkin lymphoma).

This patient had sepsis due to ventilator-associated pneumonia during the period of thrombocytopenia. However, it was found that platelets tended to decrease before this condition. In addition, the activated partial thromboplastin time (aPTT), prothrombin time (PT), and international normalized ratio (INR) were 22.2 seconds, 12.6 seconds, and 1.13, respectively. These results were within the normal range. Therefore, it could exclude disseminated intravascular coagulation.

When exploring other potential causes of thrombocytopenia in this patient, it was determined that the results of the anti-HIV and anti-HCV tests were negative. Additionally, the patient reported no history of chronic drinking. Analysis of the peripheral blood smear test revealed normochromic and normocytic red blood cells, indicating the absence of folate or vitamin B12 deficiency. Furthermore, his physical examination and medical history revealed no signs or symptoms of autoimmune disorders or lymphoproliferative diseases.

After considering all potential factors, it was determined that the patient's thrombocytopenia was drug-induced. Possible culprit medications include phenytoin and ceftazidime. The patient did not receive medication that had a potential drug interaction with phenytoin. The concentration of phenytoin in his serum was 8.8 mg/L. Liver function was still within the normal range. The multidisciplinary team decided to discontinue phenytoin and continue ceftazidime because the platelet count had decreased prior to the initiation of ceftazidime.

**Figure 1:**
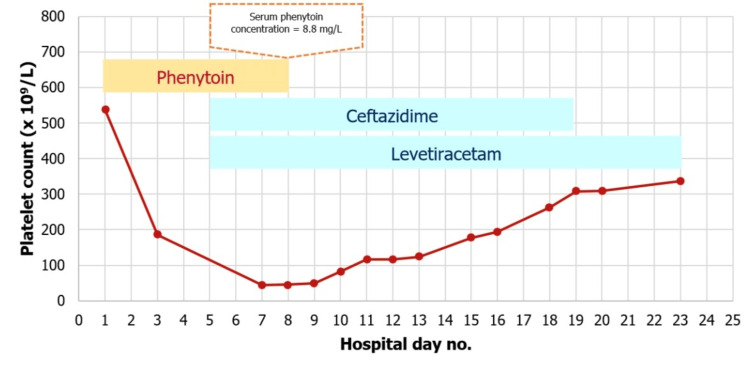
Relationship between platelet count and drug administration

After discontinuing phenytoin, the patient was gradually recovering, although he continued to receive ceftazidime. Seven days after stopping phenytoin, his daily platelet count improved from 44 to 50, 82, 116, 117, 124, and 177 x 10^9^/L, respectively. He was transferred to the medical-respiratory care unit for observation. His seizure was controlled with levetiracetam 2000 mg/day. Over time, his platelet count rose to 337 x 10^9^/L by day 15 after stopping phenytoin (Figure 1). The Naranjo algorithm score of 7 was at a probable level for phenytoin-induced thrombocytopenia.

## Discussion

Thrombocytopenia can be caused by several factors, such as infectious illnesses, hypersplenism due to chronic liver disease, chronic alcohol abuse, nutrient deficiencies, autoimmune disorders, lymphoproliferative diseases, pregnancy, and DIT [2]. Drugs are one of the most common causes of thrombocytopenia, particularly in critically ill patients. DIT has been reported in approximately 19-25% of all patients. Several medications are associated with this condition, such as acetaminophen, ampicillin, cimetidine, carbamazepine, heparin, ibuprofen, naproxen, phenytoin, piperacillin, quinine, sulfonamides, valproic acid, and vancomycin [1-3]. In this case report, we present a case of thrombocytopenia induced by phenytoin in a patient with tonic-clonic seizures.

There have been reports of phenytoin-induced thrombocytopenia in patients who received phenytoin for the treatment or prevention of seizure disorders. Commonly, phenytoin-induced thrombocytopenia begins 1-90 days after administration, and the time of platelet recovery is 3-21 days after phenytoin discontinuation [4,6-13].

Treatment of DIT involves discontinuation of the offending drug. Typically, the platelet count begins to recover after four or five half-lives of the causative drug or drug metabolite. Patients with severe thrombocytopenia and hemorrhage can receive large doses of intravenous immunoglobulin [4].

Our patient presented with severe thrombocytopenia (platelet level was 44 x 10^9^/L) seven days after receiving phenytoin. This patient was treated by discontinuing phenytoin and controlling his seizures with levetiracetam. Because there was no hemorrhaging, intravenous immunoglobulin and platelet transfusions were unnecessary for the patient. His platelet count increased to over 150 x 10^9^/L seven days after phenytoin was discontinued. This is similar to the previous case report. Previous case reports are summarized in Table 1.

**Table 1:** Summary of case reports of phenytoin-induced thrombocytopenia in the literature

Study	Patient	Medications	Platelet level (x 10^9^/L)	Onset	Management	Time of platelet recovery
Wong et al., 1985 [9]	A 67-year-old male received phenytoin for seizure prophylaxis.	Phenytoin 400 mg/day (serum phenytoin level 10.6 mg/L), dexamethasone 40 mg/day, cimetidine 1,200 mg/day	1	5 days	Both phenytoin and cimetidine were discontinued. Dexamethasone was continued throughout his hospitalization.	6 days
Brown and Chun, 1986 [10]	A 15-year-old male with tonic-clonic seizure	Phenytoin 450 mg/day (serum phenytoin level 54 mg/L)	15	2 weeks	Two doses of phenytoin were withheld and decreased dose to 300 mg/day.	1 week
Al Ghamdi et al., 2020 [4]; Yue et al. 1987 [11]	A 67-year-old male	Phenytoin 300 mg/day, dexamethasone 16 mg/day, cimetidine 800 mg/day	35	3 days	Phenytoin and cimetidine were discontinued immediately.	2 weeks
Al Ghamdi et al., 2020 [4]; Yue et al. 1987 [11]	A 22-year-old male	Phenytoin 300 mg/day, dexamethasone 16 mg/day, cimetidine 800 mg/day	28	5 days	Phenytoin and cimetidine were discontinued. Received 12 units of platelet transfusion.	2 weeks
Arbiser et al., 1993 [7]	A 81-year-old female	Phenytoin, dexamethasone 16 mg/day, cimetidine 1,200 mg/day	28	1 day	Phenytoin and cimetidine were discontinued. Received 12 units of platelet transfusion.	4 days
Holtzer et al., 1997 [8]	A 36-year-old female with generalized tonic-clonic seizure	Phenytoin 300 mg/day (serum phenytoin level 22 mg/L), dexamethasone 16 mg/day	6	5 days	Phenytoin was discontinued. Received 36 units of platelet transfusion.	10 days
Thorning and Raghavan, 2007 [6]	A 66-year-old female received phenytoin for seizure prophylaxis.	Phenytoin 300 mg/day, dexamethasone 16 mg/day	2	5 days	Phenytoin was discontinued. Received platelet transfusion.	3 days
Lau et al., 2018 [12]	A 20-year-old female received phenytoin for seizure prophylaxis.	Phenytoin 300 mg/day, dexamethasone 16 mg/day	47	2 weeks	Phenytoin was discontinued. Received 7 units of platelet transfusion.	3 weeks
Al Ghamdi et al., 2020 [4]	A 40-year-old male received phenytoin for seizure prophylaxis.	Phenytoin 300 mg/day, dexamethasone 16 mg/day	26	5 days	Phenytoin was discontinued. Received 17 units of platelet transfusion and 34 g of intravenous immunoglobulin (IVIG) over 5 days.	5 days
Gangadaran et al., 2023 [13]	A 45-year-old female	Phenytoin 300 mg/day	15	3 months	Phenytoin was discontinued. Received 7 units of platelet transfusion.	2 weeks
The present case report	A 63-year-old male with generalized tonic-clonic seizure	Phenytoin 300 mg/day	44	7 days	Phenytoin was discontinued.	7 days

DIT can be separated into two types by pathophysiology. The first type is non-immune DIT. It is defined as a direct cytotoxic effect of the drug molecules on platelets, which leads to either dysfunctional thrombopoiesis in the bone marrow or increased platelet destruction in the bloodstream [1]. These drugs include chemotherapy drugs, ganciclovir, and thiazide diuretics [4]. Another type is drug-induced immune thrombocytopenia (DITP), caused by drugs such as quinine, penicillin, sulfonamide antibiotics, and heparin [1,3,4].

The potential mechanism of phenytoin-induced thrombocytopenia is DITP. It has been hypothesized that arene oxide (epoxide), a metabolite of phenytoin, covalently bonds to platelet walls, thereby inducing antiplatelet antibodies against the hapten formed and resulting in the destruction of circulating platelets [4,6,7]. Drugs that enhance the concentration of phenytoin epoxide, such as cimetidine and dexamethasone, may be a contributing factor in phenytoin-induced thrombocytopenia [6-9].

## Conclusions

In conclusion, phenytoin-induced thrombocytopenia is a rare but serious hematological complication. It should be recognized early, particularly in patients with a high risk of hemorrhage or concurrently with medications that increase phenytoin epoxide. Since thrombocytopenia is asymptomatic unless it is severe, patients with high-risk factors may require serial complete blood counts to detect early deterioration in the platelet count.
